# Power spectral density analysis of physiological, rest and action tremor in Parkinson’s disease patients treated with deep brain stimulation

**DOI:** 10.1186/1743-0003-10-70

**Published:** 2013-07-08

**Authors:** Tjitske Heida, Eva Christine Wentink, Enrico Marani

**Affiliations:** 1 Department of Electrical Engineering, Mathematics and Computer Science, MIRA Institute for Biomedical Engineering and Technical Medicine, Biomedical Signals and Systems group, University of Twente, Enschede, The Netherlands

**Keywords:** Parkinson’s disease, Rest tremor, Action tremor, Kinetic tremor, Physiological tremor, Power spectral density, Deep brain stimulation

## Abstract

**Background:**

Observation of the signals recorded from the extremities of Parkinson’s disease patients showing rest and/or action tremor reveal a distinct high power resonance peak in the frequency band corresponding to tremor. The aim of the study was to investigate, using quantitative measures, how clinically effective and less effective deep brain stimulation protocols redistribute movement power over the frequency bands associated with movement, pathological and physiological tremor, and whether normal physiological tremor may reappear during those periods that tremor is absent.

**Methods:**

The power spectral density patterns of rest and action tremor were studied in 7 Parkinson’s disease patients treated with (bilateral) deep brain stimulation of the subthalamic nucleus. Two tests were carried out: 1) the patient was sitting at rest; 2) the patient performed a hand or foot tapping movement. Each test was repeated four times for each extremity with different stimulation settings applied during each repetition. Tremor intermittency was taken into account by classifying each 3-second window of the recorded angular velocity signals as a tremor or non-tremor window.

**Results:**

The distribution of power over the low frequency band (<3.5 Hz – voluntary movement), tremor band (3.5-7.5 Hz) and high frequency band (>7.5 Hz – normal physiological tremor) revealed that rest and action tremor show a similar power-frequency shift related to tremor absence and presence: when tremor is present most power is contained in the tremor frequency band; when tremor is absent lower frequencies dominate. Even under resting conditions a relatively large low frequency component became prominent, which seemed to compensate for tremor. Tremor absence did not result in the reappearance of normal physiological tremor.

**Conclusion:**

Parkinson’s disease patients continuously balance between tremor and tremor suppression or compensation expressed by power shifts between the low frequency band and the tremor frequency band during rest and voluntary motor actions. This balance shows that the pathological tremor is either on or off, with the latter state not resembling that of a healthy subject. Deep brain stimulation can reverse the balance thereby either switching tremor on or off.

## Introduction

Tremor at rest is, next to rigidity, akinesia or bradykinesia and postural instability, generally considered as one of the cardinal features of Parkinson’s disease (PD). It is the most common and easily recognised symptom of the disease, it is almost always prominent in the distal part of an extremity, and disappears with action and during sleep. Rest tremor generally has a frequency of about 4–6 Hz [1-4]. It may occur unilaterally or bilaterally, with the latter showing similar frequencies on both sides, but lacking a side-to-side coherency [5,6]. Frequency dissociation between upper and lower extremity tremors was also found to prevail [6]. Less well recognized in PD, but often more disabling is the occurrence of action tremor, which is any tremor that is produced during voluntary muscle contraction [3,7]. Action tremor frequency is usually reported to be slightly higher than the frequency of rest tremor, i.e. about 4–9 Hz [8,9].

With the use of tremor rating scales Louis et al. [10] observed that the action tremor score (Washington-Heights-Inwood Genetic Study of Essential Tremor Rating Scale) was associated with the rest tremor score (Unified Parkinson’s Disease Rating Scale), and suggested that both rest and action tremor are a manifestation of the underlying basal ganglia pathophysiology. According to their results neither the action nor the rest tremor score was associated with the scores for rigidity and bradykinesia, from which they hypothesized that Parkinson’s tremor may represent a different underlying pathophysiological process than the other symptoms. In contrast, according to Findley et al. [3] and Wenzelburger et al. [9] a different pathophysiology of oscillations during motion must be considered compared to the generation of tremor at rest, and they hypothesized that action tremor is an exaggeration of physiological tremor. In differentiating between rest and action tremor Carboncini et al. [11] propose the concept of pathological oscillators of central origin [12], which can be differentially recruited according to the behavioural condition. They conclude that the inability to suppress the activity of pathological oscillator(s) responsible for the action tremor plays a fundamental role in the bradykinesia associated with PD [11].

Deep brain stimulation (DBS) seems to have a similar effect on rest and postural tremor [12-15], with the latter type of tremor also referred to as a re-emergent rest tremor during postural tasks, but considered to be a form of action tremor [7]. On average it was found that deep brain stimulation (DBS) in the subthalamic nucleus (STN) reduces (rest) tremor amplitude and increases tremor frequency to values that are closer to those observed in normal physiological tremor [13,14,16,17]. Sturman et al. [14] pose that a decrease in regularity demonstrates that DBS actually changes the time-dependent structure of tremor rather than suppressing the amplitude of the pathological oscillations. In general, STN DBS operates with different magnitudes of clinical efficacy based on the specific motor deficit, but its effect may also be task-specific [14,18-20]. These differential effects of DBS on PD motor symptoms are hardly explained in literature.

The aim of this study is to explore if rest and action tremor react in a differential way to clinically effective and less effective STN DBS using quantitative methods. With action tremor we mean the tremor occurring during voluntary movement, which is also termed kinetic tremor [7]. When hypothesizing that action tremor is an exaggeration of physiological tremor, suppression of action tremor by stimulation may be expected to also have an effect on the characteristic 8–12 Hz central component, associated with physiological tremor [7,21-23]. Furthermore, will clinical effective DBS cause the characteristics of normal physiological tremor to reappear at rest? The power spectral density function of angular velocity signals recorded at hands and feet during rest and a simple tapping movement will be used to investigate the distribution of movement power over the frequency bands associated with movement, pathological and physiological tremor. Suppression of tremor by DBS is expected to result in a redistribution of movement power.

## Methods

### Subjects

A total of 7 patients participated in the study (average age 63±6.5 years, see Table 1). All except one patient received bilateral DBS (Medtronic 3389 electrode lead) in the STN; surgery took place at least three months prior to the test, and all patients satisfied the following criteria:

•Good and fast (within 5 min.) response to the stimulation;

•No major fluctuations in the symptoms due to medication;

•Good physical condition and able to fully cooperate during the experiments;

•No dementia and/or dyskinesia diagnosed during DBS treatment.

**Table 1:** Patient details (time in years)

**Pat.**	**Sex**	**Age**	**Disease dur.**	**Time after surg.**	**Targ.**	**DBSon**	**DBS80%**	**DBSoff**
1	F	68	15	6	R	2.0V, 60 μs, 140 Hz, 4-C+	1.6V	off
2	M	62	16	6	R	3.6V, 60 μs, 140 Hz, 1-C+	2.9V	off
					L	3.9V, 60 μs, 140 Hz, 5-C+	3.1V	off
3	M	61	17	1	R	3.0V, 60 μs, 145 Hz, 1-C+	2.4V	off
					L	2.8V, 60 μs, 145 Hz, 1-2-C+	2.2V	off
4	F	62	6	3	R	2.5V, 60 μs, 145 Hz, 1-C+	2.0V	off
					L	3.2V, 60 μs, 145 Hz, 1-2-C+	2.6V	off
5	F	75	13	1	R	3.5V, 120 μs, 145 Hz, 1-2+3-	2.8V	off
					L	3.3V, 120 μs, 145 Hz, 1-2+3-	2.6V	off
6	M	62	12	7	R	4.2V, 90 μs, 140 Hz, 7-C+	3.4V	off
					L	3.6V, 60 μs, 140 Hz, 0-1-2-C+	2.9V	off
7	M	54	18	6	R	3.4V, 60 μs, 140 Hz, 1-2-C+	2.7V	x
					L	4.0V, 90 μs, 140 Hz, 6-7-C+	3.3V	x

Abbreviation: *F* Female, *M* Male, *R* Right STN, *L* Left STN, *C* stimulator case, *x* not included in the experiment. Stimulation sites are indicated by 0, 1, 2, 3 corresponding to the four electrode contacts of the DBS lead on the left side, and 4, 5, 6, 7 on the right side in case of a single stimulator. Stimulation sites are indicated by 0, 1, 2, 3 for both sides in case separate stimulators for the left and right STN were used.

Medications were not withheld before the measurement session. All procedures conformed to the Declaration of Helsinki and were approved by the Medical Ethical Committee of the Medisch Spectrum Twente in Enschede, the Netherlands. All subjects signed informed consent in advance.

### Data acquisition

Four inertial sensors (MT9®, Xsens Technologies BV, Enschede, the Netherlands) measuring the angular velocity, were taped on hands and feet and connected to the Xbus master (MT9®) placed around the waist; data was sent to a laptop via Bluetooth. All signals were filtered by a 20 Hz pre-sampling filter and sampled at 50 Hz.

### Tests

Two tests were performed by each patient:

Rest tremor test. While sitting at rest the patient was reading a text aloud for 45 seconds.

Action tremor test. A tapping movement was performed as fast as possible for 30 seconds. This test was subsequently performed by the right hand, left hand, right foot, left foot. During hand tapping the wrist rested on the edge of the table; during foot tapping the heel rested on the floor.

Each test was repeated four times with different stimulation settings applied during each repetition:

DBSon. DBS settings normally used by the patient;

DBS80%. Stimulation amplitude is reduced to 80%;

DBSoff. Stimulator off.

Table 1 summarizes the settings for each patient. The order of the tests (i.e. the sequence of right/left hand, right/left foot) was randomized for each series and the order of the series (DBSon, DBS80%, DBSoff) was randomized for each patient. In between the series patients had 5 minutes of rest to adjust to the changed DBS setting.

The quantitative measures used in this study were compared to a subset of the motor examination of the Unified Parkinson’s Disease Rating Scale (UPDRS items 20 (rest tremor), and 21 (action tremor), and items 24 to 26 to score hand and foot movements) at each DBS setting. The performance of these tests was videotaped and afterwards scored by an experienced movement disorder neurologist who was blind to stimulator settings. The UPDRS scores are summarized in Table 2.

**Table 2:** UPDRS scores

**Pat.**	**DBS setting**	**UPDRS**				
		**20**	**21**	**24**	**25**	**26**
1	DBSon	0	1	x	x	x
	DBS80%	0	1	x	x	x
	DBSoff	0	1	x	x	x
2	DBSon	0	3	2	3	3
	DBS80%	0	3	2	2	2
	DBSoff	0	3	2	2	3
3	DBSon	0	0	1	1	1
	DBS80%	0	1	2	1	1
	DBSoff	0	0	2	1	1
4	DBSon	x	2	2	x	x
	DBS80%	2	2	2	2	4
	DBSoff	4	4	2	3	4
5	DBSon	0	0	3	2	2
	DBS80%	0	0	2	1	x
	DBSoff	0	0	2	2	2
6	DBSon	2	1	2	1	1
	DBS80%	3	1	2	2	2
	DBSoff	4	1	3	2	2
7	DBSon	0	0	3	3	3
	DBS80%	0	1	2	3	4
	DBSoff	0	x	x	x	2

20: rest tremor upper extremities; 21: action tremor upper extremities;

24, 25: hand movements; 26: foot movements.

### Data analysis

All analyses were performed in Matlab (the MathWorks, Inc., 2010). Prior to the analyses all recordings were high-pass filtered with a cut off frequency of 0.25 Hz (2nd order non-causal Butterworth filter).

#### Classification of tremor and non-tremor windows

**Figure 1:**
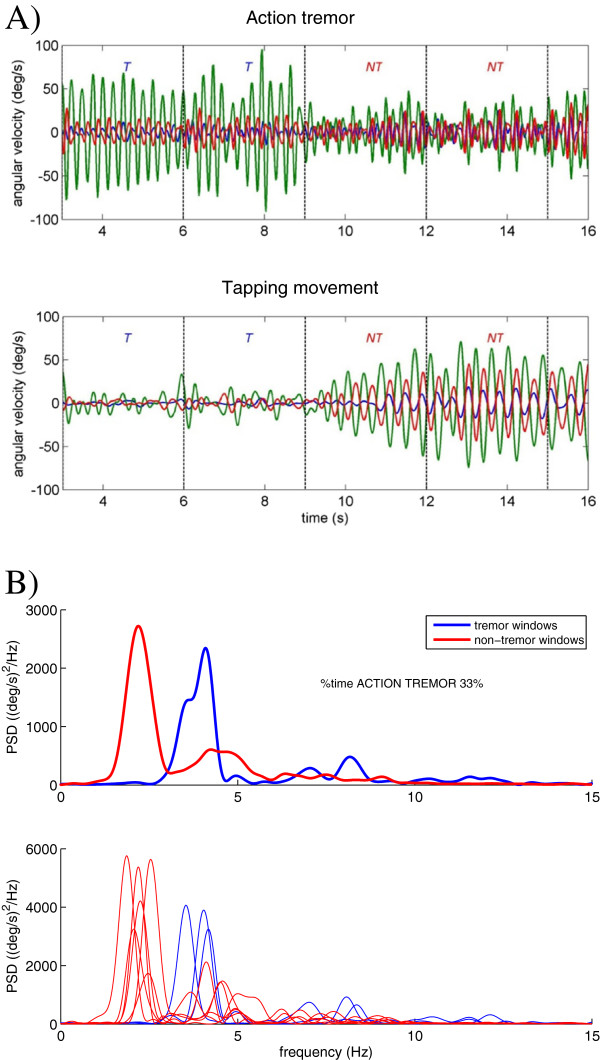
**Division of the recorded angular velocity signal from the hand of one of the patients during the action tremor test (setting DBS80%), into tremor and non-tremor windows. A)** A segment of 13 seconds of the angular velocity signals (blue: pitch, green: roll, and red: yaw) divided into an action tremor component (upper graph) and a tapping component (lower graph). The tremor component and tapping component are visualized by filtering the recorded signal: a band pass filter (4th order non-causal Butterworth filter, pass band 3.6-7.5 Hz) was used to retrieve the tremor component; filtering the signal with a low pass filter (4th order non-causal Butterworth filter with a cut off frequency of 3.4 Hz) revealed the tapping component. Classification of the 3-second windows into tremor (*T*) and non-tremor (*NT*) is indicated. **B)** Upper graph: the average power spectral density of all 3-second windows classified as tremor windows (blue line) and non-tremor windows (red line). The double peak in the power spectral density of the tremor windows expresses the variation in tremor frequency with time. Lower graph: the PSD of each 3-second window classified as tremor (blue line) or non-tremor (red line) window.

All signals were divided into windows of 3 seconds and each window was classified as a tremor or non-tremor window using an algorithm based on the method developed by Salarian et al. [24]. For each 3-second window the power spectral density (PSD) was estimated using an all-pole 6th degree autoregressive model using the Burg method. The AR model enables the detection of resonance peaks that express the oscillatory behavior of a system. The pole with highest amplitude within the frequency band of 3.5-7.5 Hz was selected as the dominant pole. Windows were classified as tremor windows when the dominant pole of one of the three axes of rotation exceeded a threshold of 0.88. The tremor frequency band and the threshold were selected based on the visual inspection of all 3-second windows of all patients. The threshold of 0.88 allows variations in tremor frequency and amplitude as normally observed in PD patients. The PSD was calculated for each of the 3-second windows (using a Hann window) over a frequency range up to 15 Hz. Figure 1 shows an example of the classification of tremor and non-tremor windows. Figure 1 B shows the average PSD over all tremor (blue line) and non-tremor (red line) windows in the upper graph; the lower graph shows the PSD of each of the 3-second windows. It can be observed in the latter graph that the classification of the tremor windows is based solely on the dominancy of the oscillatory behavior as a ‘system’ property of the extremity irrespective of the amplitude. Windows classified as non-tremor windows can still contain tremor peaks; similarly, tremor windows can also contain movement peaks.

#### Power distribution

To analyze the distribution of power of the recorded signals, three frequency bands were defined:

•<3.5 Hz, the low frequency band, associated with voluntary movements (in normal subjects voluntary movements do not occur at a rate greater than 200/min (3.3 Hz), which is expected to be lower in PD patients due to bradykinesia and rigidity [25]);

•3.5-7.5 Hz, the tremor frequency band of rest and action tremor;

•7.5-15 Hz, a high frequency band, associated with normal physiological tremor.

For each patient the relative power in each of these frequency bands for each 3-second window was calcu-lated by dividing the absolute power in the respective frequency band by the total power in the window (*P*_15,n_), i.e., the power in the range from 0 to 15 Hz. The average relative power in each of the frequency bands for the tremor (*P*_*T*_) and non-tremor (*P*_*NT*_) windows was calculated as

(1) $$P_{T,x}=\frac{1}{N_{T}}\sum_{n=1}^{N_{T}}\frac{P_{x,n}}{P_{15,n}}$$

(2) $$P_{\mathrm{NT},x}=\frac{1}{N_{\mathrm{NT}}}\sum_{n=1}^{N_{\mathrm{NT}}}\frac{P_{x,n}}{P_{15,n}}$$

with *x* indicating one of the three frequency bands as defined above, *P*_*x,n*_ the absolute power in the respective frequency band for window *n*, *P*_15,n_ the total power within the window, and *N*_*T*_ and *N*_*NT*_ the number of tremor and non-tremor windows, respectively.

The average tremor frequency within the tremor windows was determined by averaging the peak frequencies found in the PSD in case a dominant pole was found in the tremor band. For the non-tremor windows the mean frequency in the tremor band was calculated. Also, for the low and high frequency band the mean frequency was calculated for the tremor as well as for the non-tremor windows. The mean frequency for window *n* within frequency band *x* was calculated according to

(3) $$mf_{x,n}=\frac{1}{P_{x,n}}\sum_{k=1}^{N_{x}}f\left(k\right)S_{\mathrm{yy}}\left(k\right)$$

with *N*_*x*_ the number of samples of the PSD in frequency band *x*, *S*_*yy*_(*k*) the power of sample *k* with *f*(*k*) the accompanying frequency.

### Statistics

For each test and for each of the stimulation settings the average of each parameter was calculated for all four extremities of an individual patient, for the tremor and the non-tremor windows separately. Wilcoxon’s two-tailed rank-sum test with a significance level of 5% (p<0.05) was used to compare the different conditions. Bonferroni correction was applied for multiple comparisons (n=4). Spearman’s rank correlation coefficient was determined to test for correlations among extremities and for correlations between the power levels in the three frequency bands. In the scatter plots linear trend lines were determined using a robust fitting method [26].

## Results

**Figure 2:**
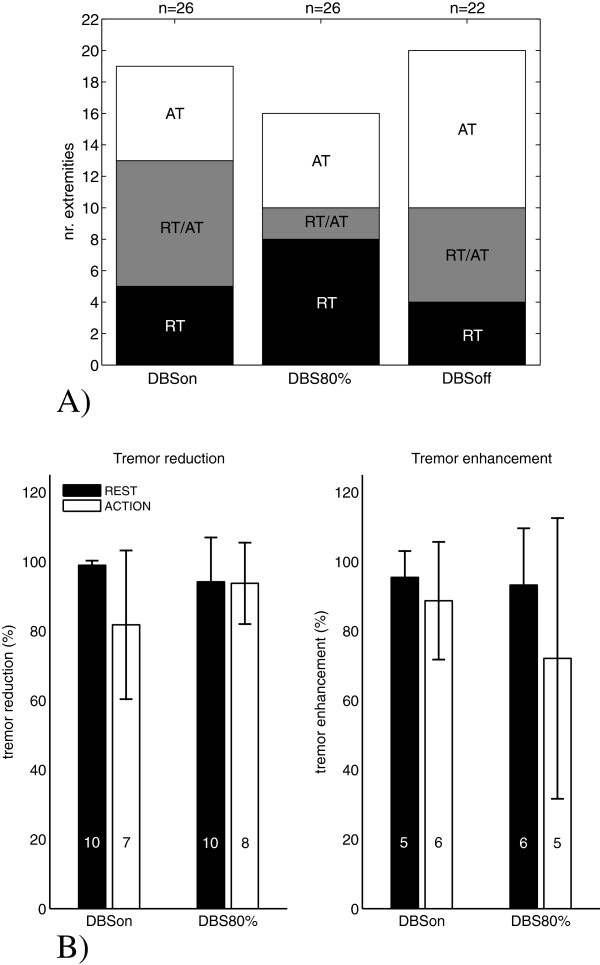
**Occurence of rest and action tremor and the effect of DBS on tremor power. A)** The number of extremities showing tremor. An extremity was included in this graph when at least one of the 3-second windows of the recorded signal at this extremity was classified as a tremor window. For each DBS setting the number of extremities showing either action tremor (AT) or rest tremor (RT), or both, is indicated. **B)** Comparing the absolute power in the tremor frequency band at DBSon and DBS80% with DBSoff a reduction or enhancement of tremor can be observed for both action and rest tremor. For this calculation the average absolute tremor power over all tremor windows for each extremity is included irrespective of the duration of tremor. In case tremor is reduced for DBSon (or DBS80%), the reduction is calculated as (P_DBSoff_-P_DBSon_)/ P_DBSoff_*100%; in case tremor is enhanced at DBSon (or DBS80%) compared to DBSoff, the enhancement is calculated as (P_DBSon_-P_DBSoff_)/ P_DBSon_*100%. The number of extremities that was included in the calculation of the average reduction or enhancement is indicated inside each bar.

All patients included in the study showed tremor. Figure 2A shows the number of extremities showing tremor, i.e. at least one of the 3-second windows of the recorded signal at an extremity was classified as tremor window. Each bar shows the number of extremities showing either action tremor (AT) or rest tremor (RT), or both, for one of the three DBS settings. On average (including all extremities at all DBS settings) rest tremor was present during 62±34% of the measurement period; action tremor was present during 54±26% of the measurement period. At an individual basis the tests showed that the effects of stimulation, in absolute sense, were different for each patient and also differential effects for hands and feet were observed. In terms of absolute power at DBSon and DBS80% a reduction of tremor or enhancement of tremor compared to DBSoff could be observed. These results are shown in Figure 2B. The average reduction or enhancement is around 90%.

### Power distribution patterns: tremor presence versus tremor absence

**Figure 3:**
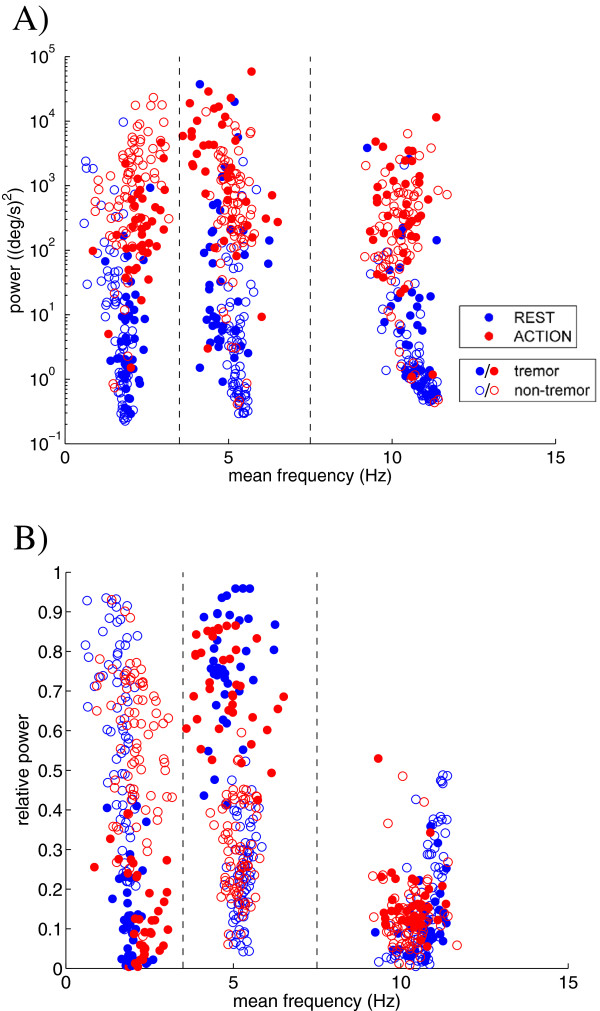
**The power-frequency relationship within the frequency bands.** The absolute **(A)** and relative **(B)** power of the angular velocity signals as a function of the mean frequency in the low frequency band (<3.5 Hz), the pathological tremor band (3.5-7.5 Hz), and the normal physiological tremor frequency band (7.5-15 Hz) for the tremor (closed markers) and non-tremor (open markers) windows of the rest tremor test (i.e. rest tremor; blue markers) and the action tremor test (i.e. the tapping movement and action tremor; red markers), respectively. Each marker represents a single extremity of an individual patient at a particular setting of the stimulator. Note that the absolute power in figure **A** is plotted on a logarithmic scale. According to the sensor specifications the power of sensor noise is around 0.0025 (deg/s)^2^/Hz, and thus recorded signals were well above noise level.

Figure 3 shows the scatterplots of the absolute (A) and relative (B) power as a function of the mean frequency in the three frequency bands for both tests (blue: rest tremor test; red: action tremor test); each marker represents one of the four extremities of a single patient at a single setting of the stimulator. Whereas the absolute power in the three frequency bands for the tremor and non-tremor windows show significant overlap (Figure 3A), a clear distinction between tremor and non-tremor windows is seen in the distribution of power over the three frequency bands (Figure 3B). Since no relatedness was found for the extremities using the Spearman correlation coefficient, the data from all extremities were combined for further analyses.

**Figure 4:**
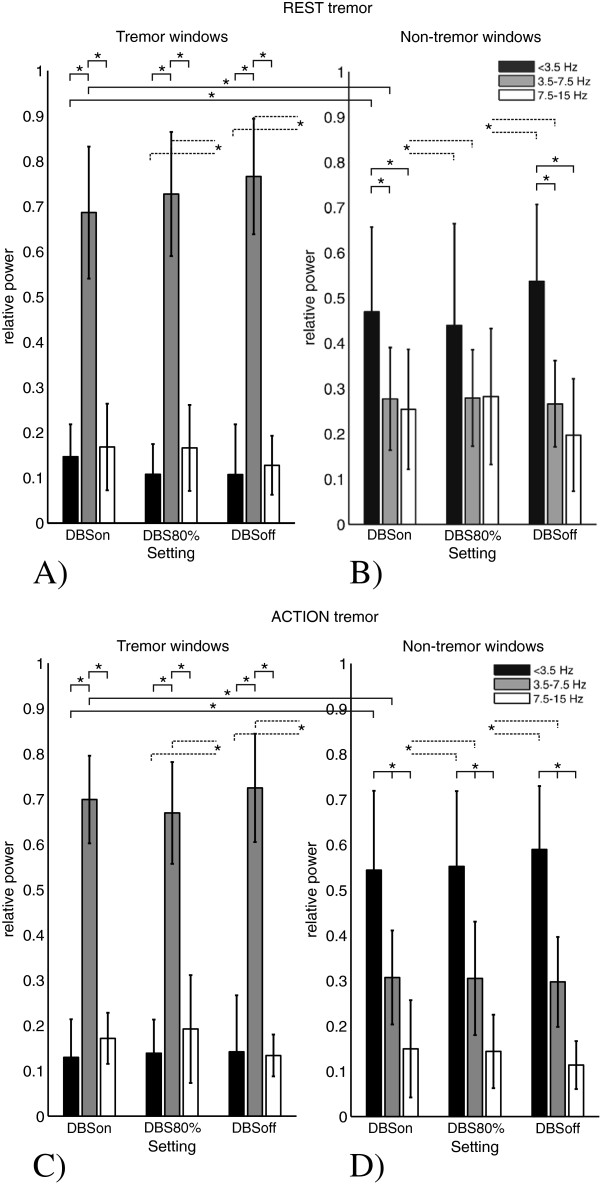
**Power distribution within tremor and non-tremor windows.** The relative power of the angular velocity signals in the low, tremor and high frequency band for the tremor **(A)** and non-tremor **(B)** windows of the rest tremor test and for the tremor **(C)** and non-tremor windows **(D)** of the action tremor test, respectively. The graphs show the average results for the group of patients; standard deviations are included. All statistically significant differences (p<0.01) between the three frequency bands for each setting as well as the comparison of tremor and non-tremor windows are indicated for each frequency band and each setting (for legibility, the significant differences between the tremor and non-tremor windows for DBS80% and DBSoff are indicated by broken lines). It must be noted that during tremor presence the power concentrated in the high frequency band was partially the result of the higher harmonic(s) of tremor (Figure **A** and **C**). However, tremor patterns rather closely resembled sinusoids (see Figure 1**A**), and therefore harmonic components were small. No significant differences were found for the power in the high frequency band when comparing tremor and non-tremor windows.

Figure 4 shows the mean and standard deviation of the relative power within the three frequency bands of the patient group for the different settings of the stimulator, for the tremor windows (A and C) and non-tremor windows (B and D). Clearly, two general patterns can be observed that are independent of the stimulation setting, but are solely determined by the absence or presence of tremor. As expected, in presence of rest or action tremor most power was contained in the tremor band; the relative power in the low and high frequency band was significantly lower (p<0.01). The relative tremor power was comparable for rest and action tremor (around 70%). Both tests showed that tremor became more dominant when present more often and/or for longer periods of time: when tremor was continuously present about 80% of the total power was concentrated in the tremor band. In absence of tremor most power was contained in the low frequency band irrespective of the resting or tapping condition.

### Low and high frequency band versus tremor band

#### Mean frequency

Both tremor types showed an average tremor frequency of 4.8 Hz. The mean frequency in the low frequency range was not related to tremor frequency under resting conditions, but the tapping rate (i.e. the mean frequency in the low frequency band) was found to be inversely related to action tremor frequency during the performance of the action tremor test (p<0.05). The tapping rate thus increased with decreasing action tremor frequency, while lower tremor frequencies are associated with slightly higher levels of relative tremor power. The mean frequency in the high frequency range was independent of tremor frequency during rest and voluntary movement.

#### Power distribution

**Figure 5:**
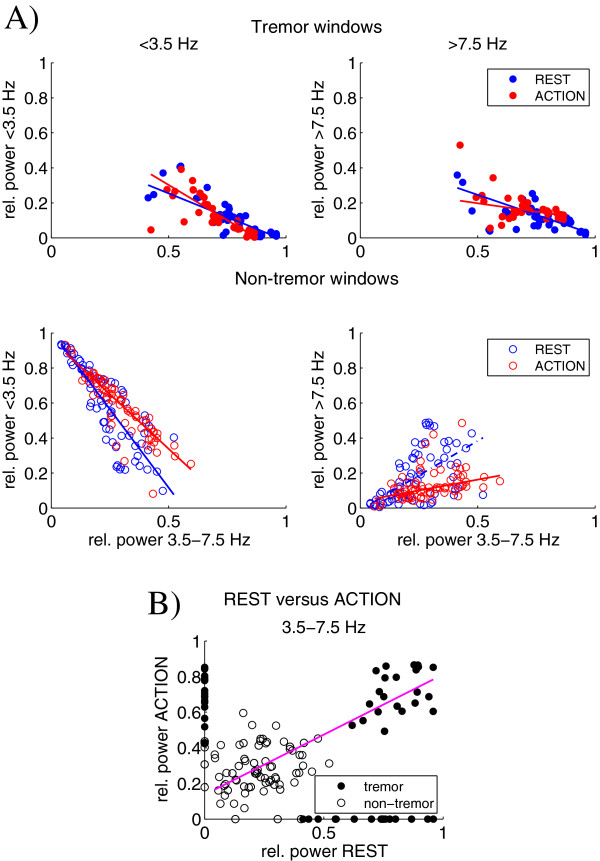
**Power exchange between the three frequency bands. A)**. Scatter plots of the relative power in the low (left panel) and high (right panel) frequency band as a function of the relative power in the tremor band, for the tremor windows (upper plots), and non-tremor windows (lower plots) of the rest tremor test (blue markers) and the action tremor test (red markers). Linear regression lines are included for the complete data set of the rest tremor test (blue line) and the action tremor test (red line) (p<0.05). Combining the results of both tests resulted in the scatter plot **(B)** showing the relation between the relative power during rest (x-axis) and action (y-axis). Each marker represents one of the four extremities of a single patient for one of the DBS settings. While each hand or foot may show either rest or action tremor, in those instances that an extremity showed both tremor types, the relative power of rest and action tremor were related as indicated by the linear regression line (p<0.05).

Figure 5 shows the scatter plots revealing the relations between the relative power in the low and high frequency band, and the relative power in the tremor band for both tests. When tremor was present (upper plots) the relative power in both the low and high frequency band decreased with an increase of relative power in the tremor band. Tremor started to become apparent when about 50% of the total power was concentrated in the tremor band. For the non-tremor windows (lower plots), a decrease in low frequency power was accompanied by an increase in high frequency power.

Each extremity may show either rest or action tremor, or both. In case an extremity showed rest as well as action tremor, the relative tremor power was found to be related (Figure 5B) (p<0.05 for the robust linear fit as indicated in the figure; Spearman’s rank correlation coefficient: ρ=0.68, p<0.01). The results of Spearman’s test for relatedness between the relative and absolute power in the low frequency and high frequency band compared to the power in the tremor band are presented in Table 3. A high negative correlation was found between the relative power in the low frequency band and the power in the tremor band for both the tremor and non-tremor windows. For the absolute power, however, these relations show a positive correlation.

**Table 3:** Spearman’s rank correlation coefficients

**Relative power**	**Rest tremor test**	**Action tremor test**
tremor windows		
<3.5 Hz vs. tremor band	ρ= −0.80, p<0.01	ρ= −0.83, p<0.01
>7.5 Hz vs. tremor band	ρ= −0.62, p<0.01	ρ= −0.33, p<0.05
non-tremor windows		
<3.5 Hz vs. tremor band	ρ= −0.88, p<0.01	ρ= −0.91, p<0.01
>7.5 Hz vs. tremor band	ρ= 0.60, p<0.01	ρ= 0.55, p<0.01
**Absolute power**	**Rest tremor test**	**Action tremor test**
tremor windows		
<3.5 Hz vs. tremor band	ρ= 0.87, p<0.01	ρ= 0.77, p<0.01
>7.5 Hz vs. tremor band	ρ= 0.93, p<0.01	ρ= 0.97, p<0.05
non-tremor windows		
<3.5 Hz vs. tremor band	ρ= 0.91, p<0.01	ρ= 0.90, p<0.01
>7.5 Hz vs. tremor band	ρ= 0.95, p<0.01	ρ= 0.95, p<0.01

### Comparison to UPDRS scores

The trends observed for the quantitative measures of the tremor windows of the rest tremor test as a function of the UPDRS scores showed that an increase in absolute and relative tremor power was expressed by a higher UPDRS score (p<0.05). Similar to the findings of Elble et al. [27] the absolute power of the angular velocity signal in the tremor frequency band was logarithmically related to the 5-point rating scale. Tremor duration also showed an increasing trend with increasing UPDRS scores, however this was not statistically significant (p>0.05). No relation to the UPDRS score was found for the quantitative measures of the action tremor test.

## Discussion

Classification of the measurement data into tremor and non-tremor windows revealed the highly intermittent character of rest and action tremor in Parkinson’s patients. Even though DBS may improve tremor, tremor may still appear for short periods of time. However, DBS may also cause worsening of tremor. Despite the large variations within the group of patients and the differential effects of stimulation on the extremities of individual patients, the distribution of the power of the recorded angular velocity signals over the low (<3.5 Hz), tremor (3.5-7.5 Hz) and high (7.5-15 Hz) frequency band showed two consistent patterns. A pattern corresponding to tremor presence, for which most power was contained in the tremor band, and a pattern corresponding to tremor absence, with most power contained in the low frequency band irrespective of the resting or tapping condition. Although the relative power in the tremor band was reduced, the latter pattern did not correspond to that of a healthy person [7,12,22,23]. The similarity in power distribution patterns, the fact that both tremor types showed the same tremor frequency (around 4.8 Hz), and the relatedness between the relative power of rest and action tremor when an extremity showed both tremor types support the suggestion of Teräväinen et al. [28], that the pathophysiology of action tremor may be similar to that of rest tremor.

We found from the power spectral density distribution of the movement signals recorded at the upper and lower extremities of Parkinson’s disease patients, that the absolute power in the tremor band during episodes without dominant rest tremor can be large. Still, in this condition the power in the low frequency band is dominating (see Figure 2A, blue markers). It seems that the relatively large power in the low frequency band balances tremor power and prevents tremor to become dominant (see the positive and negative correlation coefficients for the absolute and relative power, respectively, as presented in Table 3). In case of the action tremor test, the power in the low frequency band is the result of the performed tapping movement. A large relative power in the low frequency band, whether occurring during rest or during motor performance, may effectively compensate tremor and could occur both when stimulation was on and off; it was thus not a direct consequence of stimulation.

When rest tremor was present and became more severe the relative power in the low frequency band decreased, which may imply that compensation is starting to fail. A power concentration of 50% within the tremor band seemed to be the breaking point in this process. A large absolute power in the tremor band is not a prerequisite for a dominant tremor; tremor is not represented by a distinct resonance peak in the power spectral distribution. Likewise, a relatively low tremor power is not a prerequisite for non-dominant tremor.

Comparing UPDRS scores with the quantitative measures found for the tremor windows showed that a higher UPDRS rest tremor score is associated with a higher absolute and relative tremor power (p<0.05), and a higher absolute power in the low frequency band (p<0.05), but this score showed an inverse trend with the relative power in the low frequency band (p>0.05). The balance between the low frequency band and the tremor band seems to be the key in tremor appearance or suppression.

In advanced stages of the disease, tremor may influence the onset of voluntary movements and therewith slow down the movement, while the tremor oscillations may attract voluntary repetitive movements [29-32]. From the movement registrations during the action tremor test we found that hand or foot tapping was hindered when action tremor started to become more dominant (see Figure 1A and B). The relative tapping power (i.e. the relative power <3.5 Hz) decreased with increasing relative power in the tremor band when tremor was present or absent. A balance between the power in the low frequency range associated with movement, and tremor power is either in favor of performing movements or in generating tremor. Similar to the results of the rest tremor test, a power concentration of 50% within the tremor band seemed to be the breaking point in this process. These findings support the hypothesis that the inability to suppress the activity of pathological oscillator(s) responsible for the action tremor may play a fundamental role in akinesia associated with PD [4,33-37]. Thus, voluntary movement may suppress rest tremor if the power in the low frequency band starts to dominate (>50%) due to the movement. In contrast, movement may be inhibited if the power in the tremor band is dominating. This can also be concluded from the negative correlation between the relative power in the low frequency band and the relative power in the tremor frequency band (Table 3).

The tapping rate showed a slightly increasing trend with increasing relative tremor power, while tremor frequency slightly decreased with increasing relative tremor power, which may express a (weak) attraction between tremor and repetitive voluntary movement [31]. There was thus no slowing of the movement, which may explain the fact that no correlation between the quantitative measures of the action tremor test and UDPRS scores for bradykinesia were found.

Since during voluntary movement different pathways through the basal ganglia are activated compared to the resting state [38], the occurrence of rest and action tremor may mainly be determined by the pathways involved in different brain states and the involvement of these pathways in the degenerative processes. From the relatedness between rest and action tremor it is likely that pathways may overlap, and that both tremor types share a common pathophysiology. The relationship between neuronal activity patterns in the parkinsonian basal ganglia-thalamocortical and the cerebello-thalamo-cortical loop, and the occurrence and severity of rest and action tremor as well as the relationship with the other motor symptoms merits further investigation through simultaneous recordings in different nuclei and movement registration, possibly combined with functional imaging techniques.

It is hypothesized that by applying high frequency deep brain stimulation (DBS) the pathological neural activity patterns are overridden [19,39,40]. Stimulation of those areas in the STN that show cortically coherent oscillations at beta frequencies produces the most effective motor benefit in PD patients [30]. In recent studies, it was hypothesized that the stimulation induced suppression of the pathological central oscillators allowed the normal physiological tremor oscillations to become more dominant in the system [16]. Our results, however, showed that stimulation may suppress (or enhance) the pathological tremors, which is expressed by a shift of movement power from the tremor frequency band to the low frequency band (or vice versa). Beneficial stimulation protocols did not restore the power distribution of the recorded signals to that of a healthy subject.

Depending on the location and extent of the neurodegenerative processes in the parkinsonian brain, each extremity may be affected differently and in a non-uniform way by the disease. Electrical activation of the associated neuronal pathways will then also show differential effects. In addition, the location of the electrode will be different for each patient, and also the effect of stimulation on neighbouring structures may affect the clinical outcome. Despite these aspects and whether or not DBS suppresses or enhances tremor and/or suppresses or enhances compensation mechanisms, two consistent power distribution patterns of movement recordings were found, discerning tremor presence and absence, with the latter not resembling the power distribution pattern of a healthy person. These patterns were similar for rest and action tremor.

Although the time between changing the stimulation setting and the start of the trials was rather short, we expect that this did not have a large influence on tremor. The expected effect on tremor from STN DBS has been found to occur within seconds of the onset of stimulation [39,41]. The time it takes for symptoms to reoccur after termination of the stimulation was, however, found to vary across patients and to be related to disease duration [42]. Any underestimation of the effect of changing the settings of DBS was expected to be similar across stimulation settings allowing the comparison of the relative effects on the different stimulation settings [43].

Since the current study was set up as a pilot study, the number of subjects included was relatively small. In addition, patients were not withdrawn from medication, and the time of medication intake with respect to the measurement session may have been different for the different patients. We expect that medication may have influenced the severity and duration of tremor (both rest and action tremor), adding to the heterogeneity of the group. However, despite the small sample number and the heterogeneity of the group in absolute terms (i.e. tremor occurrence and severity, and the response to DBS), the results show a very homogeneous pattern regarding the power distribution of the movement signals in the low frequency band and the tremor band. This balance shows that tremor is either on or off; similarly, DBS can either switch tremor on or off (see Figure 2B).

## Conclusions

The distribution of power of angular velocity signals recorded from upper and lower extremities of Parkinson’s disease patients showed two general patterns, irrespective whether the patient was at rest or performing voluntary movements. During those periods that tremor was present most power was contained the tremor frequency band (3.5-7.5 Hz). When tremor was absent the lower frequencies (<3.5 Hz) dominated. Even under resting conditions a low frequency component became prominent, which seems to act as a compensation mechanism. It is hypothesized that the balance between the low frequency band and the tremor band is the key in tremor appearance or suppression with a power concentration of 50% within the tremor band the breaking point in this process. Application of deep brain stimulation resulted in a re-distribution of power in the tremor and the low frequency band, but left the parkinsonian power distribution patterns corresponding to tremor absence and presence intact.

## Competing interests

The authors declare that they have no competing interests.

## Authors’ contributions

TH was involved in the conception of the research project, has made substantial contributions to the design an execution of the data and statistical analyses, and has been involved in drafting and reviewing the manuscript. ECW was involved in the design of the study and carried out the measurements, reviewed and criticized the data and statistical analyses as well as the draft versions of the manuscript. EM was involved in reviewing and criticizing the conception and execution of the research, the data and statistical analyses, and critical revising the draft versions of the manuscript. All authors read and approved the final manuscript.
